# Two cases of aldosterone and cortisol producing adenoma with different histopathological features: A case report

**DOI:** 10.1097/MD.0000000000030008

**Published:** 2022-08-12

**Authors:** Hongjiao Gao, Li Li, Haoming Tian

**Affiliations:** a Department of Endocrinology and Metabolism, West China Hospital of Sichuan University, Chengdu, Sichuan, China; b Department of Endocrinology and Metabolism, The Third Affiliated Hospital of Zunyi Medical University (The First People’s Hospital of Zunyi), Zunyi, Guizhou, China; c Institute of Clinical Pathology, West China Hospital of Sichuan University, Chengdu, Sichuan, China.

**Keywords:** adrenal tumor, cosecretion, Immunohistochemistry, primary aldosteronism, subclinical Cushing’s syndrome

## Abstract

**Rationale:**

Primary aldosteronism (PA), including aldosterone and cortisol producing adenoma (A/CPA), is the most common trigger of secondary hypertension. The prevalence of A/CPA may be higher than what we could recognize previously with similar studies, but only a few relevant immunohistochemical reports have confirmed this information. Collecting more clinical features and immunohistochemistry data may help us to understand A/CPA, which is very important for avoiding misdiagnosis and improving outcomes in patients with A/CPA.

**Patient concerns:**

Both individuals included in this study had hypertension for >10 years. Computed tomography scans revealed the presence of adrenal nodules 1 year ago in patient A and 10 months ago in patient B (based on the date of the final version of this report). The relevant clinical features support PA accompanied by subclinical Cushing syndrome.

**Diagnosis:**

Aldosterone and cortisol producing adenoma.

**Interventions:**

The adrenal adenoma on the affected side was removed and pathological examination and immunohistochemistry were performed. Both the patients received short-term hydrocortisone treatment.

**Outcomes:**

The blood pressure of both patients improved after surgery. Cytochrome P450 (CYP)11B1, CYP11B2, parathyroid hormone receptor 1 (PTH1R), calcium-sensing receptor (CaSR), and vitamin D3 receptor (VD3R) were all positively expressed, but the histopathological features of the expression region differed.

**Lessons:**

The occurrence of A/CPA may be related to calcium metabolism disorders. For A/CPA, the diversity in immunohistochemistry suggests many uncertainties regarding the pathogenesis of the disease. A/CPA should be considered in new clinical and pathological classifications of PA to gain more attention from the medical community.

## 1. Introduction

According to previous population studies, about 5 to 10% of patients diagnosed with hypertension have primary aldosteronism (PA),^[1]^ which is the most common cause of secondary hypertension. As the understanding of such conditions deepens in the medical community, PA has been divided into 6 subtypes based on etiology.^[2]^ Complementarily, unilateral PA has been divided into 6 subtypes based on histopathological features.^[3]^ However, the prevalence of aldosterone and cortisol producing adenoma (A/CPA) is higher than what we have previously recognized, and A/CPA may also become one of the subtypes of PA in the future. To date, there are few reports addressing this issue and studies available in the community lack immunohistochemical results. Here, we reported 2 cases of A/CPA with different histopathological features and reviewed the literature concerning this specific condition to further understand the clinical aspects of such cases.

## 2. Case report

### 2.1. Case 1

A 53-year-old Han Chinese woman who had been suffering from hypertension for 13 years reported that a doctor found adrenal nodules in her body 1 year prior to her admission to our hospital. She was administered amlodipine besylate (5 mg QD) for blood pressure control. On admission, her body mass index (BMI) was 29.21 kg/m^2^, and blood pressure was 145/98 mm Hg. The plasma aldosterone concentration (PAC) and direct renin concentration (DRC) were determined using an automatic chemiluminescence instrument (Diasorin Liaison XL) from the Italian company Solin and its aldosterone and renin concentration detection kit. The PAC reference interval for upright position was 3.0 to 35.0 ng/dl, and DRC was 4.4 to 46.1 µIU/ml. Furthermore, the patient’s dynamic postural test showed that PAC in supine position was 21.3 ng/dL, DRC was 0.98 µIU/mL, plasma aldosterone to direct renin concentration ratio (ADRR) was 21.73 (ng/dL)/(µIU/mL), upright position ALD-DRC-ADRR: 60.1 ng/dL to 2.33 µIU/mL to 25.79 (ng/dL)/(µIU/mL). Blood potassium was measured by the ion-selective electrode method (indirect method), blood potassium was measured by the colorimetric method, and serum parathyroid hormone and 25-OH-D3 were measured by chemiluminescence. The patient’s serum potassium was 3.44 mmol/L (reference range 3.5–5.3 mmol/L), serum calcium was 2.24 mmol/L (reference range 2.11–2.52 mmol/L), and serum parathyroid hormone (PTH) was 8.12 ng/L (reference range 1.60–6.90 ng/L), serum 25-OH-D3 was 35.6nmol/L (reference range 47.7–144 nmol/L). Both the saline infusion test and the captopril inhibition test showed no inhibition, supporting the diagnosis of PA. Adrenal enhanced computed tomography (SOMATOMO Definition Flash, Siemens, Germany) findings confirmed a small low-density nodule of 2.1*1.8 cm in the right adrenal gland with clear boundaries and uneven density of mild enhancement. Computed tomography scan results also showed slight atrophy of the left adrenal glands, which were uniformly enhanced. The results of adrenal venous sampling (AVS) are shown in Table 1. The patient had a right-sided A/CPA, and AVS showed that the contralateral (left) cortisol was significantly inhibited. Based on the bilateral PAC and adrenal contrast-enhanced computed tomography findings, a right adrenal lesion was identified in our patient, and the patient was treated surgically. After the resection of the right adrenal gland, pathological examination suggested a cortical adenoma of 3 cm*2.3 cm*1.7 cm, with a partially incomplete capsule, golden section (solid and medium hardness), and cortex thickness of 0.1 cm.

**Table 1:** Results of adrenal venous sampling.

	Vena cava	Left adrenal vein –1	Right adrenal vein –1
PAC (ng/dL)	35.4	2600	7280
Serum cortisol (nmol/L)	613	1775	28,236
PAC/serum cortisol	0.06	1.46	0.26

### 2.2. Case 2

A 59-year-old Han Chinese man was admitted to the hospital with symptoms triggered by a right adrenal nodule >10 months ago. He was diagnosed with hypertension more than a decade ago and was treated with amlodipine besylate (5 mg QD) for blood pressure control. On admission, the patient’s BMI was 29.21 kg/m^2^, and blood pressure was 160/102 mm Hg. His dynamic postural test showed that supine position PAC was 19.48 ng/dL, plasma renin activity (PRA) 0.33 ng/mL/h, plasma aldosterone/renin activity ratio (ARR) 59.03 (ng/dL)/(ng/mL/h), and upright position PAC-PRA-ARR 32.56 ng/dL to 1.24 ng/mL/h to 26.26 (ng/dL)/(ng/mL/h). The radioimmunoassay method was used to detect PAC using a kit from Tianjin Jiuding Medical Bioengineering Co., Ltd., and to detect PRA using a kit from Beijing North Biotechnology Research Institute Co., Ltd. The upright position reference interval for PAC was 9.8 to 27.5 ng/dL, while that for PRA was 0.93 to 6.56 ng/mL/h. The serum potassium level was 3.9 mmol/L, serum calcium level 2.25 mmol/L, PTH 5.05 ng/L, and 25-OH-D3 54.8 nmol/L. Similar to the case 1, his saline infusion test and captopril inhibition test showed no inhibition, confirming a diagnosis of PA. A computed tomography scan showed a shadowed figure of an oval soft tissue density nodule in the right adrenal gland with uniform density and regular shape that was obviously enhanced. The shadow was about 2.6 × 1.9 cm with clear peripheral fat and atrophy of the left adrenal gland. The patient underwent surgery for the direct removal of the right adrenal tumor directly without AVS. Pathological examination confirmed the presence of a cortical adenoma with considerable pigment deposition. The nodule was about 2.5 cm*2.2 cm*2 cm, with grayish-black or grayish-yellow section (solid, medium), clear boundaries with the surrounding area, and adrenal cortex thickness 0.1 to 0.3 cm.

Neither patient had a family history of high blood pressure or adrenal tumors. Neither patient had full-moon face or bloody appearance. For both patients, no fat pads were identified at the back of their neck, and no hirsutism, purple striae, and centripetal obesity were observed. Plasma metanephrine and normetanephrine were normal. The test of cortisol confirmed the absence of circadian rhythm. Overnight 1 mg dexamethasone inhibition test and classic low-dose dexamethasone suppression test showed no inhibition (Table 2), thus supporting the diagnosis of SCS.

**Table 2:** Results of serum cortisol circadian rhythm and dexamethasone suppression test in both patients.

Patient	ACTH, serum and urine cortisol at baseline				1 mg DST	LDDST	
	8:00 STC	0:00 STC	8:00 ACTH	24-h UFC	STC after test	STC after test	24-h UFC after test
1	409	137	8.87	135.9	134	125	77.8
2	478	351	1.26	295.4	99.1	511	326.6

Both the patients received short-term hydrocortisone supplementation after surgery. Their blood pressure did not return to normal, but improved significantly. PAC, DRC, ADRR, serum cortisol, and serum potassium levels were all normal 3 months after surgery.

**Figure 1.**
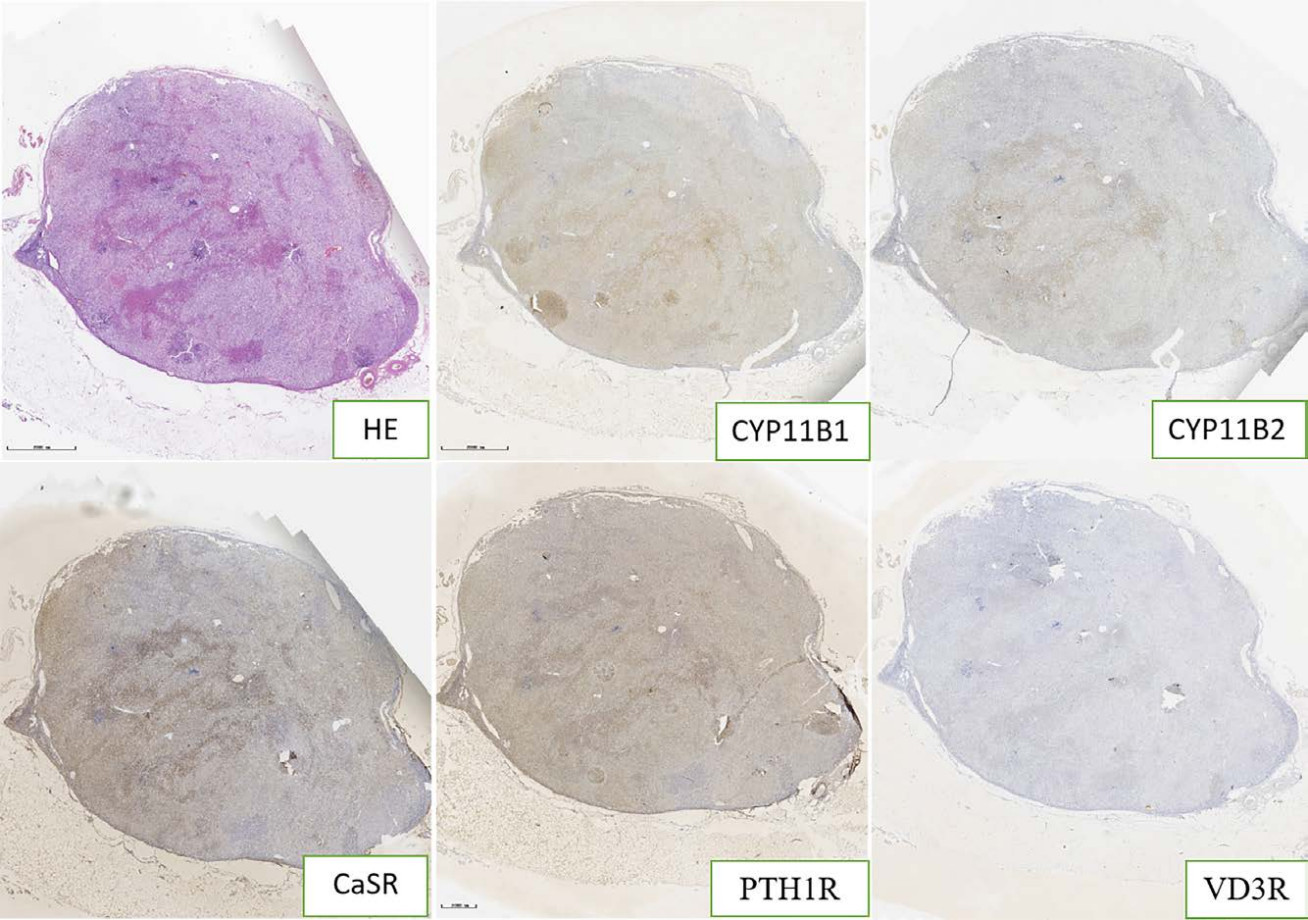
CYP11B1, CYP11B2, PTH1R, CaSR, and VD3R immunohistochemical expressions of irregular cord-like distribution in case 1.

**Figure 2.**
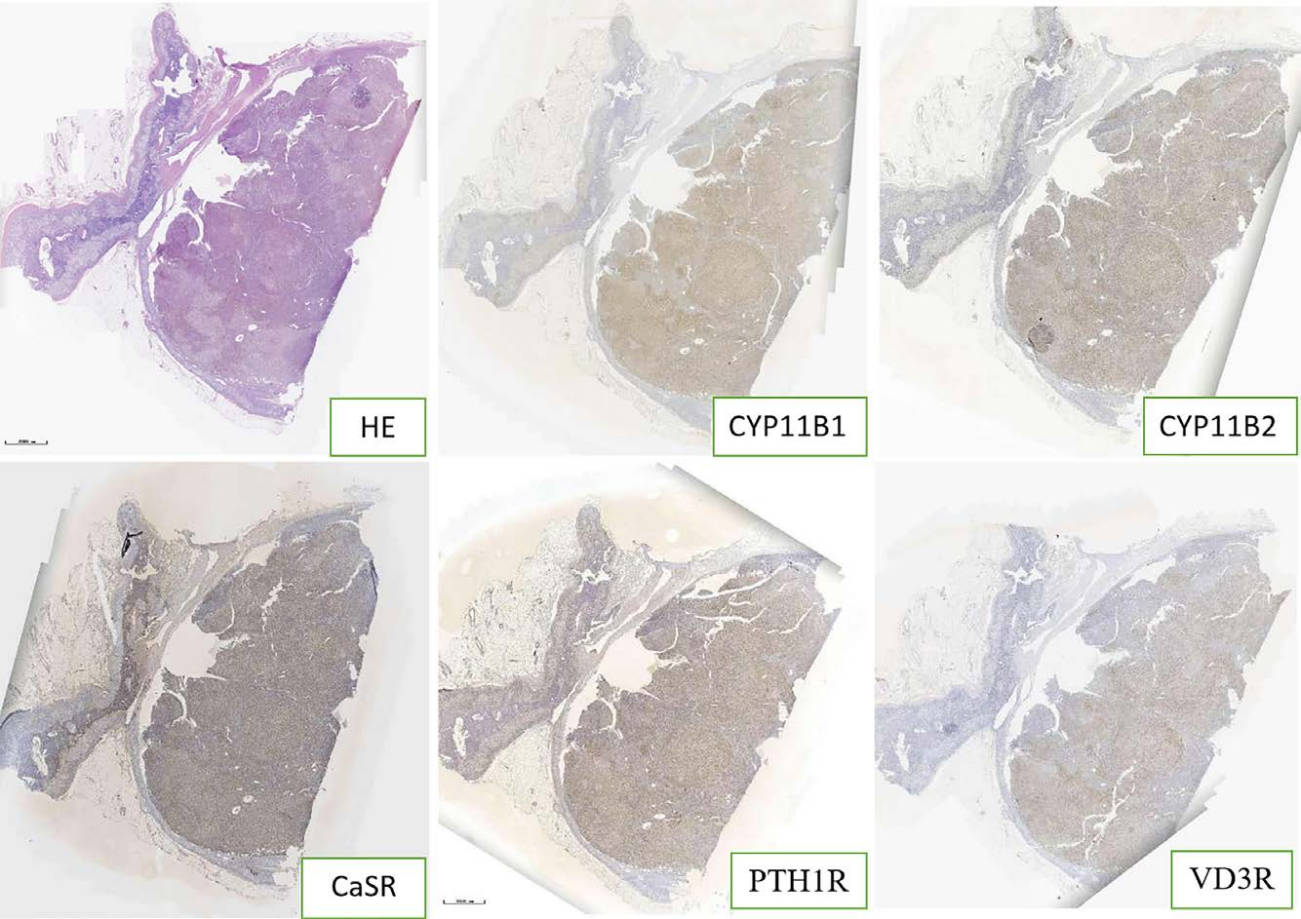
CYP11B1, CYP11B2, PTH1R, CaSR, and VD3R immunohistochemical expressions of multiple germinal centers in case 2.

**Figure 3.**
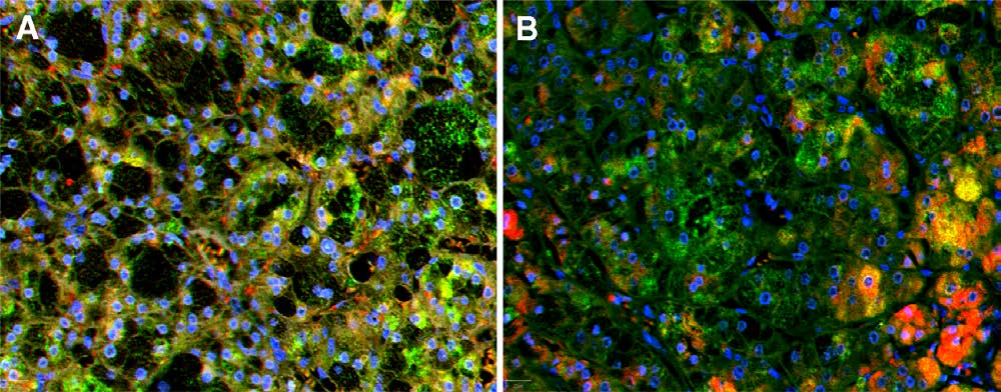
CYP11B1 (red) and CYP11B2 (green) expressions in different cells of adrenal cortical adenoma. Yellow represents the region where CYP11B1 expression overlaps with CYP11B2 expression. (A) (case 1) magnification: ×10, (B) (case 2) magnification: ×100.

**Figure 4.**
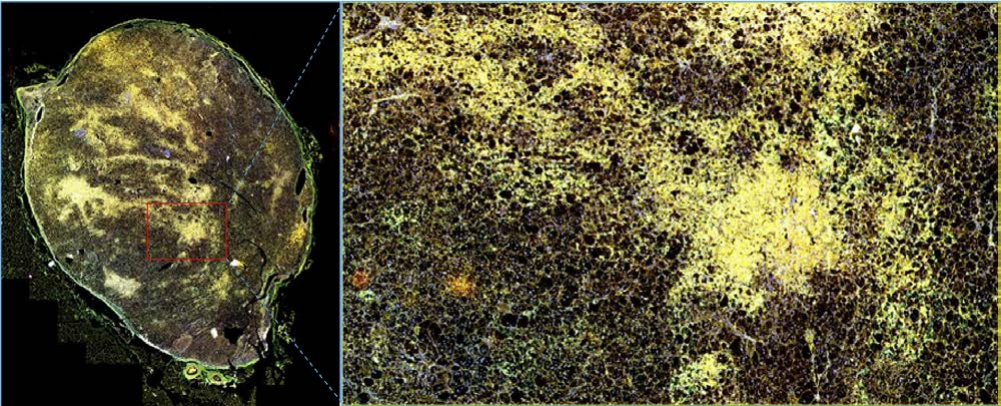
CYP11B1, CYP11B2, PTH1R, CaSR, and VD3R multiple fluorescence immunohistochemistry with overlapping distribution areas in case 1.

**Figure 5.**
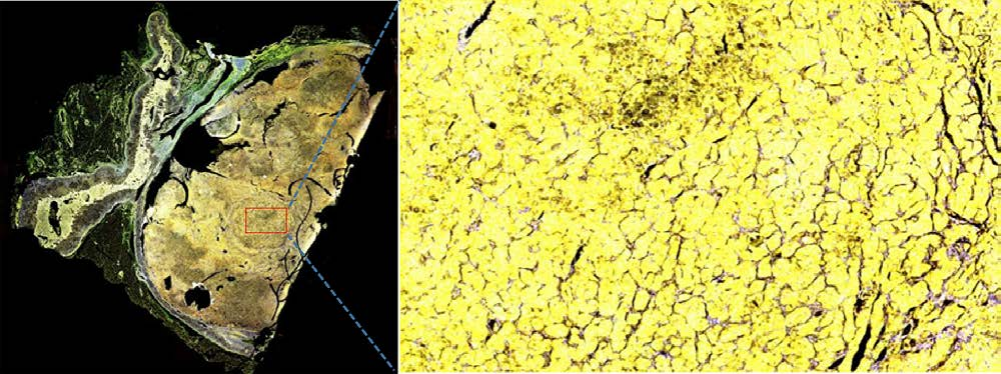
CYP11B1, CYP11B2, PTH1R, CaSR, and VD3R multiple fluorescence immunohistochemistry with overlapping distribution areas in case 2.

The expression of cytochrome P450 (CYP) 11B1, CYP11B2, parathyroid hormone receptor 1 (PTH1R), calcium-sensing receptor (CaSR), and vitamin D3 receptor (VD3R) were detected by immunohistochemistry and multiple fluorescence immunohistochemistry of the postoperative adrenal tissue in 2 patients. The paraffin section specimens were handled using the Opal Polaris 7-color Manual immunohistochemistry kit (Akoya Biosciences, USA) as the manufacturer’s recommendation. Multiple fluorescence immunohistochemistry images were acquired using the Vectra Polaris Automated Quantitative Pathology Imaging System (PerkinElmer, USA). Antibodies against CYP11B1 (MABS502) and CYP11B1 (MABS1251) were purchased from the Merck Group. Antibody against VD3R (12550) was purchased from Cell Signaling Technology. Antibody against CaSR (MA1-934) was purchased from Thermo Fisher Scientific. Antibody against PTH1R (MAB5709) was purchased from R&D Systems. The results showed that CYP11B1 (encoding 11β-hydroxylase), CYP11B2 (encoding aldosterone synthase), and Ca^[2]^ metabolism-related receptors (CaSR, VD3R, and PTH1R) were positively expressed in the same region of tumor tissue. CYP11B1 and CYP11B2 were expressed in the zona glomerulosa - and zona fasciculata-like cells, respectively. Interestingly, the expression sites of CYP11B1, CYP11B2, PTH1R, CaSR, and VD3R in both patients were heterogeneous (Figs. 1–5).

## 3. Discussion

In 1977, Hogan et al^[4]^ reported a case of PA accompanied by subclinical Cushing syndrome (SCS) for the first time, and subsequent studies found this condition was not uncommon.^[5–7]^ According to the literature concerning immunohistochemistry based on CYP11B1 and CYP11B2 staining, PA accompanied by SCS may occur in various forms, with A/CPA being the most common. Zhang et al have reported a case of bilateral adenomas that secrete cortisol and aldosterone, respectively,^[8]^ while some researchers observed diffuse staining of CYP11B1 in adenoma lesions, and high CYP11B2 expression in the adjacent adrenal cortex.^[9]^ Studies have pointed out that about 10% of PA patients have A/CPA,^[7,10,11]^ and in PA patients with a single adenoma, the prevalence of A/CPA is 9.7 to 21%.^[5,12]^ However, misdiagnosis often occurs because of the absence of clinical manifestations of overt cortisol levels increase in patients. Despite these diagnostic challenges, patients with A/CPA are indeed more prone to metabolic abnormalities^[13]^ and present a higher risk of cardiovascular events,^[14]^ a phenomenon that is worthy of attention in the medical community. A diagnosis of A/CPA may also help avoid misinterpretation of AVS^[15]^ and the risk of postoperative adrenal cortical insufficiency.^[16]^

The exact pathogenesis of A/CPA is still unclear, but its histopathological features may provide important clues for further research. Xin Gao et al^[17]^ confirmed that aldosterone-producing cells are sensitive to fluctuations in extracellular Ca^[2]^ levels, and Ca^[2]^ metabolism may directly affect hormone production, especially in the process of cosecretion of aldosterone and cortisol in the tumors of APA patients. It is noticed in this study that the expression sites of CYP11B1 and CYP11B2 in A/CPA are in the same region as those for the calcium metabolism-related receptors PTHR, CaSR, and VD3R, which is consistent with the aforementioned mechanism. In addition, different expression patterns of CYP11B1 and CYP11B2 were also identified in cases A/CPA, and it was speculated that there might be other somatic gene mutations related to calcium channels.

Furthermore, the histopathological features of A/CPA were found to exhibit zona fasciculata and zona glomerulosa-like cells. Multiple fluorescence immunohistochemical images showed that both cases 1 and 2 had coexpression of CYP11B1 and CYP11B2.

To date, in several case reports, the volume of A/CPA cases is larger than of pure APAs, and generally, the diameter of A/CPA is >2.0 or 2.5 cm.^[5,16]^ According to previous research on this matter, the minimum diameter in these cases is 1.1 cm, but no masses <1 cm in diameter have been reported.^[18]^ The diameters of A/CPA for the cases reported in this study are 2.1 cm and 2.5 cm, respectively, and it is speculated that the incidence of A/CPA in APA may be higher than that reported previously in other clinical studies. However, with the increase in tumor volume, the CYP11B1 expression level was further increased. Simultaneously, cortisol levels in the body gradually increased, confirming the diagnosis of SCS. Even in cases of confirmed A/CPA, CYP11B1 immunostaining may show low expression. However, since A/CPA is composed of many aldosterone-secreting and cortisol-secreting cells, even if the yield per cell is low, the overall number is still significant and, because of that, SCS may still occur.^[19]^

Interestingly, in both cases of A/CPA reported in our study, PAC increased by > 33% from baseline after the postural test, which is different from typical APA cases. This phenomenon may be related to the enhanced response to renin and decreased response to adrenocorticotrophic hormone in the A/CPA patients. Whether it can be used as a diagnostic clue for A/CPA should be clarified through a relevant study involving a larger sample size. Contralateral adrenal atrophy in patients with A/CPA is a concern for the future.

The first immunohistochemical analysis of the CYP11B1, CYP11B2, and calcium metabolism-related receptors (CaSR, VDR and PTH1R) of A/CPA by multiplex immunofluorescence was performed. Unfortunately, epinephrine or norepinephrine was not used to correct adrenal venous aldosterone levels in the AVS. This is the lesson that we have learned. We also failed to genetically sequence the resected adrenal tissue, which will be our future research direction.

In conclusion, the occurrence of A/CPA may be related to calcium metabolism disorders, and the diversity of A/CPA in immunohistochemistry suggests that there are many uncertainties in the pathogenesis of the disease. Therefore, A/CPA should be considered in new clinical and pathological classifications of PA to gain more attention from the medical community.

## Acknowledgments

We thank the patients for approval to publication.

## Author contributions

Conceptualization, design and writing: Hongjiao Gao, Haoming Tian.

Clinical and research management: Hongjiao Gao, Haoming Tian.

immunohistochemistry and multiple fluorescence immunohistochemistry: Li Li.

All authors read and approved the final article.

## References

[1] Funder JW Carey RM Mantero F. The management of primary aldosteronism: case detection, diagnosis, and treatment: an endocrine society clinical practice guideline. J Clin Endocrinol Metab. 2016;101:1889–916. 26934393 10.1210/jc.2015-4061

[2] Young WF Jr. Diagnosis and treatment of primary aldosteronism: practical clinical perspectives. J Intern Med. 2019;285:126–48. 30255616 10.1111/joim.12831

[3] Williams TA Gomez-Sanchez CE Rainey WE. International histopathology consensus for unilateral primary aldosteronism. J Clin Endocrinol Metab. 2021;106:42–54. 32717746 10.1210/clinem/dgaa484 PMC7765663

[4] Hogan MJ Schambelan M Biglieri EG. Concurrent hypercortisolism and hypermineralocorticoidism. Am J Med. 1977;62:777–82. 871129 10.1016/0002-9343(77)90883-x

[5] Hiraishi K Yoshimoto T Tsuchiya K. Clinicopathological features of primary aldosteronism associated with subclinical Cushing’s syndrome. Endocr J. 2011;58:543–51. 21521926 10.1507/endocrj.k10e-402

[6] Piaditis GP Kaltsas GA Androulakis II. High prevalence of autonomous cortisol and aldosterone secretion from adrenal adenomas. Clin Endocrinol (Oxf). 2009;71:772–8. 19226269 10.1111/j.1365-2265.2009.03551.x

[7] Bhatt PS Sam AH Meeran KM. The relevance of cortisol co-secretion from aldosterone-producing adenomas. Hormones (Athens). 2019;18:307–13. 31399957 10.1007/s42000-019-00114-8 PMC6797639

[8] Zhang Y Tan J Yang Q. Primary aldosteronism concurrent with subclinical Cushing’s syndrome: a case report and review of the literature. J Med Case Rep. 2020;14:32. 32075693 10.1186/s13256-020-2353-8 PMC7031945

[9] Fushimi Y Tatsumi F Sanada J. Concurrence of overt Cushing’s syndrome and primary aldosteronism accompanied by aldosterone-producing cell cluster in adjacent adrenal cortex: case report. BMC Endocr Disord. 2021;21:163. 34384396 10.1186/s12902-021-00818-2 PMC8359021

[10] Fujimoto K Honjo S Tatsuoka H. Primary aldosteronism associated with subclinical Cushing syndrome. J Endocrinol Invest. 2013;36:564–7. 23385627 10.3275/8818

[11] Lau JH Sze WC Reznek RH. A prospective evaluation of postural stimulation testing, computed tomography and adrenal vein sampling in the differential diagnosis of primary aldosteronism. Clin Endocrinol (Oxf). 2012;76:182–8. 21895732 10.1111/j.1365-2265.2011.04202.x

[12] Yang Y Xiao M Song Y. H-score of 11β-hydroxylase and aldosterone synthase in the histopathological diagnosis of adrenocortical tumors. Endocrine. 2019;65:683–91. 31332713 10.1007/s12020-019-02022-8

[13] Akehi Y Yanase T Motonaga R. High prevalence of diabetes in patients with primary aldosteronism (PA) associated with subclinical hypercortisolism and prediabetes more prevalent in bilateral than unilateral PA: a large, multicenter cohort study in Japan. Diabetes Care. 2019;42:938–45. 31010944 10.2337/dc18-1293

[14] Nakajima Y Yamada M Taguchi R. Cardiovascular complications of patients with aldosteronism associated with autonomous cortisol secretion. J Clin Endocrinol Metab. 2011;96:2512–8. 21593113 10.1210/jc.2010-2743

[15] Fallo F Bertello C Tizzani D. Concurrent primary aldosteronism and subclinical cortisol hypersecretion: a prospective study. J Hypertens. 2011;29:1773–7. 21720261 10.1097/HJH.0b013e32834937f3

[16] Späth M Korovkin S Antke C. Aldosterone- and cortisol-co-secreting adrenal tumors: the lost subtype of primary aldosteronism. Eur J Endocrinol. 2011;164:447–55. 21270113 10.1530/EJE-10-1070

[17] Gao X Yamazaki Y Tezuka Y. The crosstalk between aldosterone and calcium metabolism in primary aldosteronism: A possible calcium metabolism-associated aberrant “neoplastic” steroidogenesis in adrenals. J Steroid Biochem Mol Biol. 2019;193:105434. 31351131 10.1016/j.jsbmb.2019.105434

[18] Yasuda S Hikima Y Kabeya Y. Clinical characterization of patients with primary aldosteronism plus subclinical Cushing’s syndrome. BMC Endocr Disord. 2020;20:9. 31931803 10.1186/s12902-020-0490-0 PMC6958814

[19] Fallo F Castellano I Gomez-Sanchez CE. Histopathological and genetic characterization of aldosterone-producing adenomas with concurrent subclinical cortisol hypersecretion: a case series. Endocrine. 2017;58:503–12. 28405879 10.1007/s12020-017-1295-4 PMC5638684

